# Isolated middle mediastinal mass associated with immunoglobulin G4-related disease

**DOI:** 10.1186/s40792-021-01151-5

**Published:** 2021-03-17

**Authors:** Haruaki Hino, Noriyuki Tanaka, Hiroshi Matsui, Takahiro Utsumi, Natsumi Maru, Yohei Taniguchi, Tomohito Saito, Koji Tsuta, Tomohiro Murakawa

**Affiliations:** 1 Department of Thoracic Surgery, Kansai Medical University, 2-3-1 Shinmachi Hirakata-shi, Osaka, 573-1191 Japan; 2 Department of Pathology and Laboratory Medicine, Kansai Medical University, 2-3-1 Shinmachi Hirakata-shi, Osaka, 573-1191 Japan

**Keywords:** Immunoglobulin G4-related disease; mediastinal tumor, Systemic autoimmune disease

## Abstract

**Background:**

Immunoglobulin G4-related disease (IgG4-RD) is a multi-organ disorder predominantly occurring in middle-aged to elderly male patients characterized by multi-organ fibrosis, specific pathological findings of storiform fibrosis with IgG4-positive plasma cell infiltration, and elevated serum IgG4 level. We herein report a rare presentation of IgG4-RD forming an isolated mass in the middle mediastinum mimicking a mediastinal tumor and discuss the clinical significance of mediastinal IgG4-RD.

**Case presentation:**

An 82-year-old male patient without any symptom was referred due to left middle mediastinal mass (3.8 × 2.4 cm). Because of suspected lymphoma, Castleman’s disease, and lymphangitis due to tuberculosis, we performed a thoracoscopic resection for diagnosis and treatment. The mass was yellowish white with well-encapsulated, and storiform fibrosis with plasma cell infiltration, and obliterative phlebitis were observed microscopically. Additional immunohistochemical stain revealed IgG4-RD. Other radiological findings and serological results did not show evidence of other organs being affected from IgG4-RD nor autoimmune diseases. He is now followed at outpatient clinic without additional treatment for over a year, and an enhanced computed tomography does not show any recurrence.

**Conclusion:**

It was a rare presentation of IgG4-RD forming isolated middle mediastinal mass, which suggests that we might suspect IgG4-RD for undetermined mediastinal mass in case of middle to elderly male patient.

## Background

Immunoglobulin G4-related disease (IgG4-RD) was firstly reported as a sclerosing pancreatitis, which was characterized by a storiform fibrosis with IgG4-positive plasma cell infiltration and elevation of serum Immunoglobulin G4 [1]. Till date, it has been diagnosed as IgG4-RD in a wide variety of organs, including salivary gland, thyroid, pituitary gland, biliary system, kidney, aorta, retroperitoneum, lung, and mediastinum [2]. Based on both the clinical and pathological features, the general concept of IgG4-RD has been established as a new systemic autoimmune disease category. We herein report a rare presentation of IgG4-RD as forming an isolated mass in the middle mediastinum mimicking a mediastinal tumor, and considered the clinical significance of IgG4-RD.

## Case presentation

**Fig. 1:**
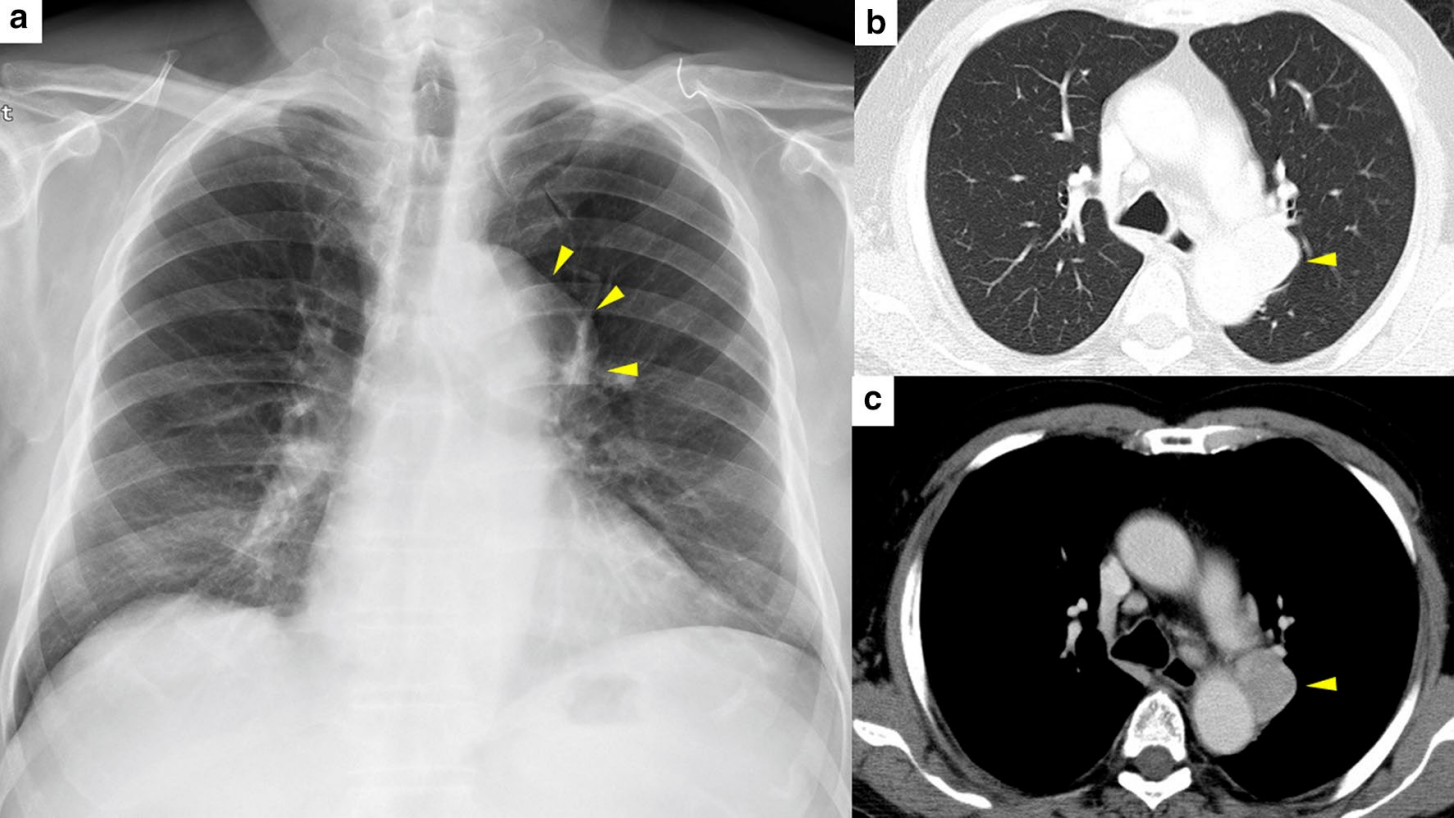
Preoperative imaging. **a** Chest X-ray showed an abnormal mass in the left mediastinum (yellow arrowheads). **b** Enhanced computed tomography of lung field (yellow arrowheads). **c** Enhanced computed tomography of mediastinal field; the left mediastinal mass was a homogenous round-shaped 3.8 × 2.4 cm in diameter (yellow arrowheads)

**Fig. 2:**
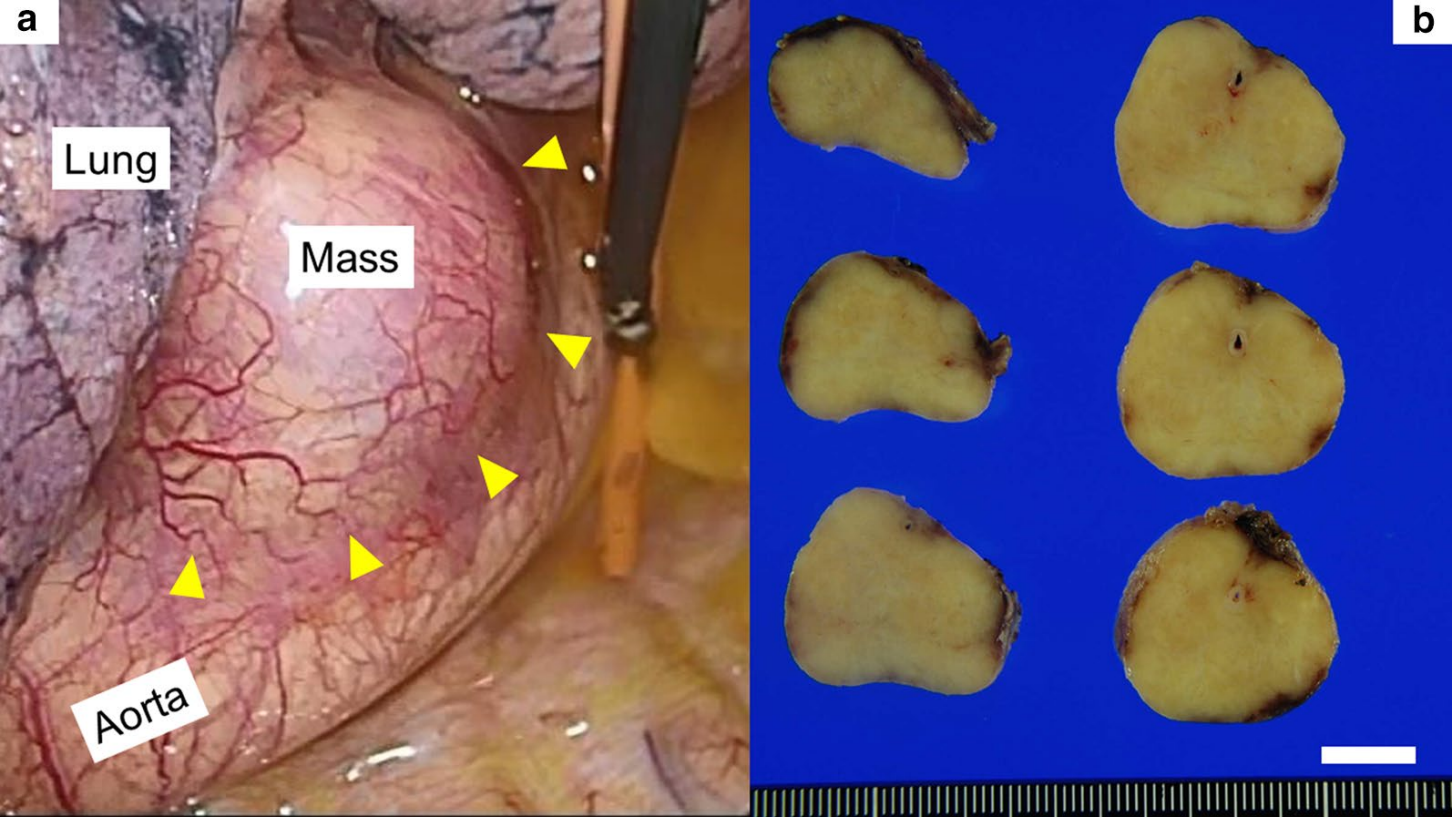
Intraoperative view of the left-side mediastinum and macroscopic findings of the mediastinal mass. **a** Intraoperative view showed that the mass was well-encapsulated neighboring to the aorta (yellow arrowheads). **b** Cross section of the mediastinal mass was elastic and homogenous yellowish; white bar = 1 cm

**Fig. 3:**
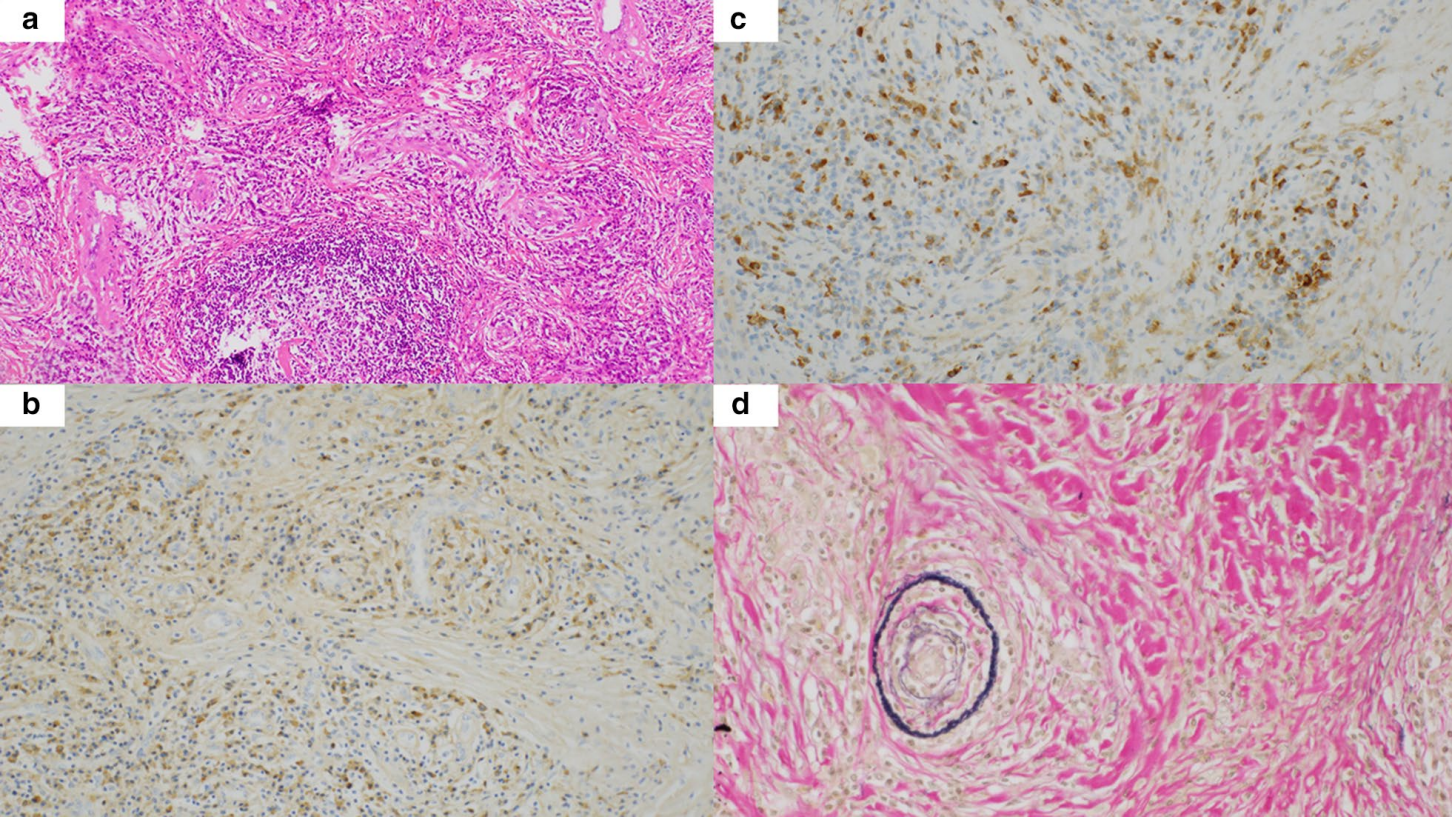
Pathological findings of the mediastinal mass. **a** Hematoxylin and eosin stain showed a fibrosis with plasma cell infiltration (× 40). **b** Immunohistochemical stain of IgG showed that IgG plasma cell infiltration was confirmed (× 200). **c** Immunohistochemical stain of IgG4 showed that IgG4-positive plasma cells were ascertained and the IgG4/IgG cell ratio was ≥ 40% (× 200). **d** Elastica van Gieson stain showed that an obliterative phlebitis was detected (× 200). *IgG* Immunoglobulin G

An 82-year-old male patient without any symptoms was referred to our institute due to an abnormal shadow detected by chest X-ray in his annual health examination. An enhanced computed tomography showed a homogenous round-shaped mass 3.8 × 2.4 cm in diameter located in left middle mediastinum (Fig. 1a–c). However, no inflammatory changes or mass lesions were detected in abdominal organs, such as the pancreas, biliary system, and lung fields in the preoperative computed tomography. Medical examination of the whole body, including neck and inguinal lymph nodes, salivary glands, and skin was performed; however, no abnormal physical findings were detected. He did not have any significant medical history, including malignancy and any other autoimmune disease. Because his serum markers of anti-acetylcholine receptor antibody, beta-human chorionic gonadotropin, alpha-fetoprotein, and soluble interleukin-2 receptor were within normal range, we suspected malignant metastatic lymph node of unknown origin, Castleman’s disease, solitary fibrous tumor, or lymphangitis due to tuberculosis. Therefore, we performed a thoracoscopic surgery to obtain a diagnosis and initiate treatment. Intraoperatively, it was an elastic encapsulated tumorous mass (Fig. 2a), which was removed from the aorta and vagus nerve with comparative ease. Operation time was 129 min and bleeding amount was 60 mL. The macroscopic cross section was yellowish white with well-encapsulated (Fig. 2b). A storiform fibrosis, lymphoid follicles with IgG4-positive plasma cell infiltration (a ratio of IgG4/IgG ≥ 40%), and an obliterative phlebitis were observed microscopically (Fig. 3a–d). Based on the above findings, we highly suspected that the pathological diagnosis was IgG4-RD. Postoperatively, we confirmed that his serum IgG, IgG4, IgA, and interleukin-6 (IL-6) levels were within the normal range. Data were as follows: IgG, 1302 (normal range, 870–1700) mg/dL; IgG4, 50 (≤ 134) mg/dL; IgA, 129 (110–410) mg/dL; and IL-6, 1.0 (≤ 4.0) pg/mL. A possible diagnosis of Castleman’s disease with elevated IL-6 level was suspected; however, the present case had normal range of IL-6.

**Fig. 4:**
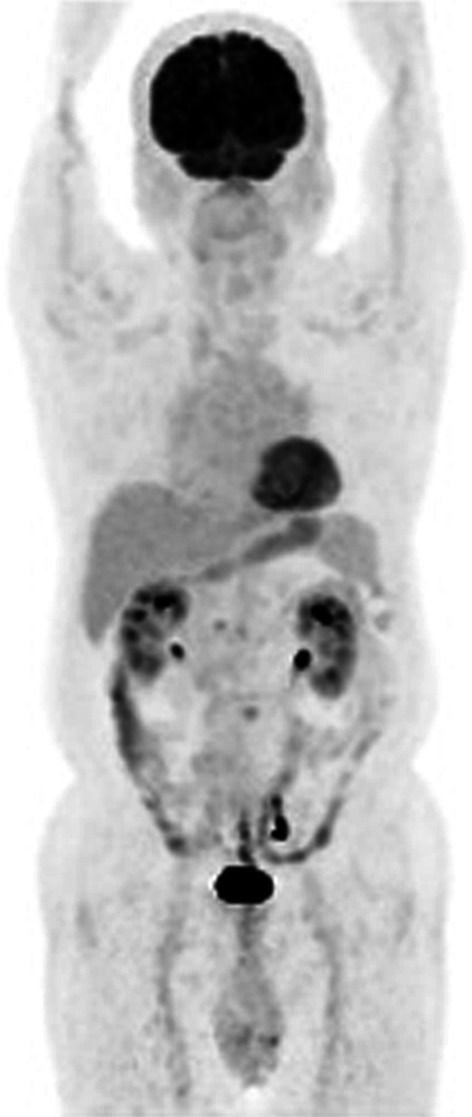
Image of F-18–2-fluoro-2-deoxy-D-glucose positron emission tomography computed tomography. No other affected organs were detected in the whole-body imaging postoperatively

We evaluated the pathological and serological findings, imaging results, and physical examinations according to the Comprehensive Diagnostic Criteria for IgG4-RD, and the patient’s condition (normal serum IgG4 level; histopathology IgG4 + /IgG + cells, > 40%; and IgG4 cells/HPS, > 10) met the criteria of a “probable” case of IgG4-RD [3]. Furthermore, we investigated the exclusion criteria of serological examination according to ACR/EULAR classification criteria for IgG4-RD [4]. Based on the criteria, additional serological findings were as follows: white blood cell, 4,200 (normal range 3500–8500/µL); platelet count, 16.0 × 10^4^ (14–34 × 10^4^); eosinophil count, 172 (21–252/µL); myeloperoxidase-anti-neutrophil cytoplasmic antibody (MPO-ANCA), < 0.5 (< 3.5 IU/mL); anti-Scl-70 antibody, < 1.0 (< 10 U/ mL); anti-Ro/SSA antibody, < 1.0 (< 10 U/mL); and anti-La/SSB antibody, < 1.0 (< 10 U/mL), which did not meet the exclusion criteria. Therefore, the diagnosis was probable IgG4-RD. He was discharged at 6 postoperative days uneventfully. He is now followed at outpatient clinic without additional treatment for over a year, and an enhanced computed tomography does not show any recurrence or any other malignancy. The survey for possible another systemic disease using F-18–2-fluoro-2-deoxy-D-glucose positron emission tomography computed tomography postoperatively did not detect other affected organs from IgG4-RD nor autoimmune diseases (Fig. 4).

## Discussion

IgG4-RD is a multi-organ affected disorder predominantly in middle to elderly male patient characterized by multi-organ fibrosis, specific pathological findings of storiform fibrosis with IgG4-positive plasma cell infiltration and elevation of serum IgG4 [1, 2]. Among patients with IgG4-RD, intrathoracic pulmonary manifestation of solid nodule, interstitial change, and mixed types with lymphadenopathy was complicated in 14.0%–27.0% cases; however, mediastinal mass formation was less except for a posterior mediastinal fibrosis in 0.8%–3.4% cases [5, 6]. IgG4-RD presenting an isolated mediastinal mass without an intrathoracic lesion was rare and 3 patients have been reported thus far. Their masses were located in anterior mediastinum (size range; 4.7–7.0 cm); therefore, thymoma, lymphoma, and teratoma were initially suspected [7–9]. Similar to our case, they were all middle to high-aged male patients (45, 58, and 71 years) without any symptoms, and had no significant elevation of serum IgG4 nor medical history of IgG4-RD, with the exception of another case report with complications due to presence of both an anterior mediastinal mass and autoimmune pancreatitis [10]. Although IgG4-RD of an anterior mediastinal mass or a posterior mediastinal fibrosis has been known to date, this is a first report of IgG4-RD forming an isolated middle mediastinal mass, suggesting that IgG4-RD could affect every location of mediastinum which has an abundant lymphatic network, and that the more number of patients underdiagnosed as IgG4-RD in mediastinum might be potentially existing in clinical practice. Because a tiny mediastinal nodule like lymph node swelling is difficult to determine the correct diagnosis histologically, any mediastinal IgG4-RD might be overlooked. Based on the above findings, we might suspect IgG4-RD for an undetermined mediastinal tumor especially in middle to elderly male patient and if suspected, serum IgG4 level and complication of extrathoracic IgG4-RD should be confirmed during pretreatment examination.

As for the etiology of IgG4-RD, a recent investigation has revealed that an imbalance of immune regulatory system, which activates Th2-dominant regulatory T cell, was associated with specific fibrotic change in affected organs [2]. However, it is still unclear whether IgG4 antibody contributes to “pathogenic effect” of driving inflammation, fibrosis, and organ damage or has merely “bystander effect” of downregulating an unknown preceding inflammation process [11]. Although the definitive etiology of IgG4-RD has not been elucidated thus far, diagnosing IgG4-RD that affects every part of the organ, including the thoracic cavity, is important and challenging [12] With regard to the differential diagnosis against IgG4-RD, multicentric Castleman’s disease, other lymphoproliferation disorders, autoimmune diseases, allergic disorders of atopic dermatitis, and asthma are considered. Based on the 2019 American College of Rheumatology criteria, diagnoses other than IgG4-RD should be excluded by confirming serological marker levels: white blood cell, platelet, and eosinophil count, IL-6, MPO-ANCA, anti-Scl-70 antibody, anti-Ro/SSA antibody, and anti-La/SSB antibody as well as levels of IgG, IgG4, and IgA. In the present case, serum markers were confirmed to be within normal limits, suggesting that we should carefully diagnose IgG4-RD by differentiating other autoimmune diseases. Additionally, in the present case, none of the abnormal clinical manifestations, including skin and respiratory conditions, were detected in the medical examination. Recently, an overlap disorder between IgG4-RD and multicentric Castleman’s disease was reported, making diagnosis of IgG4-RD more difficult [13]. In any case, a few cases of IgG4-RD presenting as a mediastinal mass have been reported to date; we might suspect more number of IgG4-RD affecting every organ, such as in mediastinal lesion with cautious consideration of other lymphoproliferation disorders or autoimmune diseases.

Considering the treatment for IgG4-RD, immunosuppressive agents, such as glucocorticoids, are quite effective in sclerosing pancreatitis and another IgG4-RD [1, 2]. In previous three cases and our case of mediastinal IgG4-RD, a mediastinal mass was completely resected; however, the utility and significance of surgical resection and the need for additional immunosuppressive therapy are unclear due to a small number of cases. Furthermore, it is reported that IgG4-RD has a potential risk of malignancies (10.4%), including lymphoma and non-lymphoid tumors; colon cancer or lung cancer, etc., which is based on an irregular immunological disorder of regulatory T cell for tumor immunity [14]. Therefore, it is recommended to examine an extrathoracic IgG4-RD and malignant disease for mediastinal IgG4-RD and also have close observation for long term.

## Conclusions

We experienced a rare presentation of IgG4-RD with mass formation in the middle mediastinum, requiring correct pathological findings and several serological examinations to distinguish from lymphoproliferation diseases and autoimmune disorders. More numbers of underdiagnosed IgG4-RD in mediastinum might be seen in clinical practice, and those case studies and longer follow-up could clarify the nature of the disease, treatment, and prognosis in future.

## Data Availability

The datasets supporting the conclusions of this article are available on reasonable request.

## Abbreviations

IgG4-RD: Immunoglobulin G4-related disease
CT: Computed tomography
