# Correction to: Isolated middle mediastinal mass associated with immunoglobulin G4-related disease

**DOI:** 10.1186/s40792-021-01168-w

**Published:** 2021-04-07

**Authors:** Haruaki Hino, Noriyuki Tanaka, Hiroshi Matsui, Takahiro Utsumi, Natsumi Maru, Yohei Taniguchi, Tomohito Saito, Koji Tsuta, Tomohiro Murakawa

**Affiliations:** 1 Department of Thoracic Surgery, Kansai Medical University, 2-3-1 Shinmachi Hirakata-shi, Osaka, 573-1191 Japan; 2 Department of Pathology and Laboratory Medicine, Kansai Medical University, 2-3-1 Shinmachi Hirakata-shi, Osaka, 573-1191 Japan

## Correction to: Surg Case Rep (2021) 7:69 https://doi.org/10.1186/s40792-021-01151-5

Following publication of the original article [1], the authors identified an error in the author name of Koji Tsuta.

The incorrect author name is: Kouji Tsuta.

The correct author name is: Koji Tsuta.

The author group has been updated above and the original article [1] has been corrected.

## References

[1] Hino  H, Tanaka N, Matsui H, Utsumi T, Maru N, Taniguchi Y, Saito  T, Tsuta K, Murakawa T (2021). Isolated middle mediastinal mass associated with immunoglobulin G4-related disease. Surg Case Rep.

